# A hybrid model to study the demographic profile of women in view of assisted reproductive techniques through machine learning models

**DOI:** 10.3389/frph.2026.1773150

**Published:** 2026-05-28

**Authors:** Latika, Renuka Arora

**Affiliations:** Department of CSE, Jagannath University, Bahadurgarh, Delhi, India

**Keywords:** ART, IVF, ML, women health, predictive modelling, hormonal imbalance, PCOD, AI

## Abstract

Assisted Reproductive Techniques (ART) refers to the reproductive measures that address the issues like infertility, low sperm count, unable to conceive and help the couples to achieve pregnancy. Various Assisted Reproductive techniques like *in-vitro* Fertilization, Intrauterine Insemination are used to treat patients with infertility. This paper studies the demographic profile of women in view of Assisted Reproductive Techniques through Machine Learning Models. Artificial Intelligence is a vast field that has proved its worth in almost all areas. Machine learning is a branch of AI where computers learn from the data provided and improve their performance by learning from the data provided. Demographic profile of Women is important to study from the view of ART methods as this would make machine understand whether the women is healthy or not. She needs to change her lifestyle or opt for Assisted Reproductive Techniques to achieve a successful conception. In this paper, Machine Learning models are applied on Primary data i.e. Data is collected from women of age 25–60 and profile of women is studied to achieve the healthy and non-healthy status of women. The study evaluates Logistic Regression, Decision Tree and Linear Discriminant Analysis, demonstrating that the hybrid approach achieves the highest accuracy (for Healthy Women: F1 score is: 0.80, Precision is: 1.00 and recall is 0.67), (for women with health issue F1 score is 0.93, Precision is 0.88 and Recall is 1.00). Further the study highlights the importance of women health and important factors that decide whether women need Assisted Reproductive Methods to achieve conception or not.

## 1 Introduction

A demographic profile of a woman is useful when considering Assisted Reproductive Techniques (ART) or IVF to achieve successful conception (1). The first IVF was conducted in 1977. Since then, the use of ART methods such as IVF and Intrauterine Insemination have been widely used to achieve pregnancy. However, these methods can be costly and have serious health effects. ART involves a lot of medications and injections where the ovaries are stimulated to produce more eggs; these eggs are then surgically retrieved from the body. After this, sperm is taken and cleaned and the eggs and sperm are mixed under a particular lab environment to produce embryos. These embryos are then transferred into the woman’s body so that embryos can plant themselves to the uterine lining and hopefully lead to a successful pregnancy. This procedure can cause significant mental and physical side effects that may affect the result of pregnancy (2, 12). Changes in lifestyle caused by technological revolutions have led many to lead a more sedentary life. Lifestyle changes have also involved changes to eating habits, sleep schedules, alcohol intake, and medicine use. These lifestyle changes, however, have affected fertility rates (3, 11–14). Infertility refers to the inability to conceive after a year of unprotected intercourse. Infertility can be caused by blocked fallopian tubes, Polycystic Ovarian Disease, Polycystic Ovary Syndrome, hormonal imbalance, and low sperm count, among other things. The complexity of infertility depends on factors such as and duration of attempts to conceive. ART techniques such as IUI and IVF provide successful conception options for infertile couples. However, both the demographic features of the woman and her lifestyle can impact on fertility. Data from the National Centre for Health Statistics (NCHS) says that approximately 8.8% of the American population is affected by infertility. As per the study conducted in United Kingdom the female infertility was found to be 12.5% and male infertility was 10.1%. There is a need to study the demographic profile of women as factors like age affect ability to conceive. There is also a need to educate women about their reproductive health and make them aware about issues their body may face that may lead to infertility (15–18).

As per the objective, the aim to is to understand the factors, such as age, BMI, and sleep schedule, that could impact on a woman’s health. To achieve this, data were collected from women aged 25–60. Data were collected from both married and unmarried women. Various supervised Machine Learning Algorithms were used to achieve this objective. A hybrid model was built to predict whether the woman is healthy or not. The features used are “Age”, “Weight”, “You Suffer from”, “Height(in cms)”, “Do u face such symptoms”, “You loose and gain weight dynamically?”, “Do you take stress?”, “Breakfast options”, “Periods Cycle is Regular?”, “BMI (click on the link to check yours)”, “Are you Ovulating?”, “You are an early bird”, “Gadgets Commitment (love to spend time on laptops and mobile for hours)”, “Do you Exercise?”, “Do you take antioxidants in your diet?”, “Do you take Alcohol?”, “Are you open to some heating environment like are you exposed to heat (like taken up an occupation of chef)”, “Do you Conceived in First Year of marriage?”, “Intercourse Frequency in a week”, “You are a junk food lover?”, “You prefer exercise or medicines to make your body fit and healthy”. In total, 21 features were used to study the demographic profiles of women. Various Supervised Machine Learning Algorithms were used like Support vector Machine, Random Forest, Decision Tree, K-Nearest Neighbors, and a hybrid model that achieved 90% accuracy.

In this research paper, we developed a hybrid predictive model using supervised machine learning models. Notably, this is the first research paper with work on primary data with 21 features to predict the health status of women. This paper informs women about their reproductive health and educates them on their health status, helping them decide whether to conceive through ART. Furthermore, if they opt for techniques such as IVF, IUI, or ICSI, this research highlights specific areas for improvement—such as stress reduction and lifestyle changes—to increase the likelihood of a successful conception.

## 2 Materials and methods

We collected data from both married and unmarried women aged 25–60. A total of 200 responses were collected, out of which 150 were retained after data cleaning and processing. The data were collected with prior permission that the data provided would only be used for research purposes, ensuring compliance to ethical standards and personal privacy. A Google Form was built under the guidance of renowned IVF specialists. We focused on different features that play a vital role in determining women’s reproductive health.

We collected data based on 30 features, out of which 21 features were selected to build a hybrid predictive model. Of the 21 features, five have numeric-valued attributes and 16 have nominal-valued attributes. The table below (Table 1) gives a brief description of each attribute, along with a brief explanation of its relevance to the demographic profile of women.

**Table 1:** Demographic profile (women).

Attribute	Description	Type
Age	Age is an important factor in the study of fertility and defining reproductive health	Numeric
Weight	Weight is major feature	Numeric
Height (in cms)	Measure to find BMI	Numeric
BMI (click on the link to check yours)	Measure to know whether your weight is in a healthy range relative to your height	Numeric
Conditions you Suffer From	It is a categorical field that has values like hyperthyroidism, PCOD, Obese, PCOS; if any problem persists it is marked with 1, if no problems it is marked with 0	Numeric
Symptoms Experienced	Categorical field with values like Healthy, Irregular Periods, pelvic or abdominal pain; if any symptoms are there it is marked as 1 else it is 0	Numeric
Weight Fluctuation	Major Factor in determining a woman’s health is if she gains or loses weight frequently (yes is marked as 1, no is marked as 0)	Numeric
Stress Levels	Major feature for various health issues like diabetes, gestational diabetes, and miscarriage (Yes is treated as 1 and No as 0)	Numeric
Breakfast Options	Categorical feature with options like “Healthy breakfast” and “no time for breakfast”. This helps to understand a person’s normal routine.	Numeric
Menstruation Regularity	Factor to know about whether she is menstruating or not (values Yes is marked with 1 and No with 0)	Numeric
Ovulation Status	To know whether she is ovulating or trying to conceive (Yes is marked with 1 and No is marked with 0)	Numeric
Early Riser Status	Factor to know whether she is an “early bird” or a morning person (Yes is marked with 1 and No is marked with 0)	Numeric
Technology Use (do you spend significant time on a laptop or mobile phone)	This assesses screen time, laptop time, use of mobile phones, or use any other electronic gadgets (Yes is marked with 1 and No is marked as 0)	Numeric
Exercise	Factor to know how healthy a women is. She exercises to maintain her health or not (Yes is marked with 1 and No is marked with 0)	Numeric
Antioxidant Consumption	This measures whether she takes supplements or medicines of any type to boost her immunity or reproductive health (Yes is marked with 1 and No is marked with 0)	Numeric
Alcohol Consumption	To know about alcohol intake as it can affect both reproductive health and fetus health (Yes is marked with 1 and No is marked with 0)	Numeric
Exposure to Heat Sources	To know whether she is exposed to heat sources such as factories, warehouses, and kitchen (high kitchen hours) with Yes marked as 1 and No marked as 0	Numeric
Conception in the First Year of marriage	To know the successful conception in the first year of marriage (Yes marked as 1 and no marked with 0)	Numeric
Weekly Intercourse Frequency	To understand whether the woman has a healthy sex life, which is important for conception	Numeric
Junk Food Consumption	To know about food intake habits (Yes marked as 1 and no is marked as 0)	Numeric
Preference for exercise or supplements for a healthy body	To know whether the woman prefers to use medicines or exercise to stay fit and healthy (yes marked with 1 and no with 0)	Numeric

In addition to standard features such as age, weight, and BMI, our study focused on novel variables including heat exposure and technology use. Both these factors affect fertility (4). Heat stress has a negative impact on the reproductive organs in human body and, in women, causes irregular mensuration cycles, hormonal imbalance, and decreased ovarian function (5). Climate change affects the chances of successful conception and has adverse effects on reproductive organs (6). The presence of electromagnetic fields also has potential effects on human reproductive organs. Higher exposure to electromagnetic fields through the overuse of technology can lead to poor sperm quality, the improper functioning of ovaries, and negative pregnancy results.

### 2.1 Proposed system

**Figure 1:**
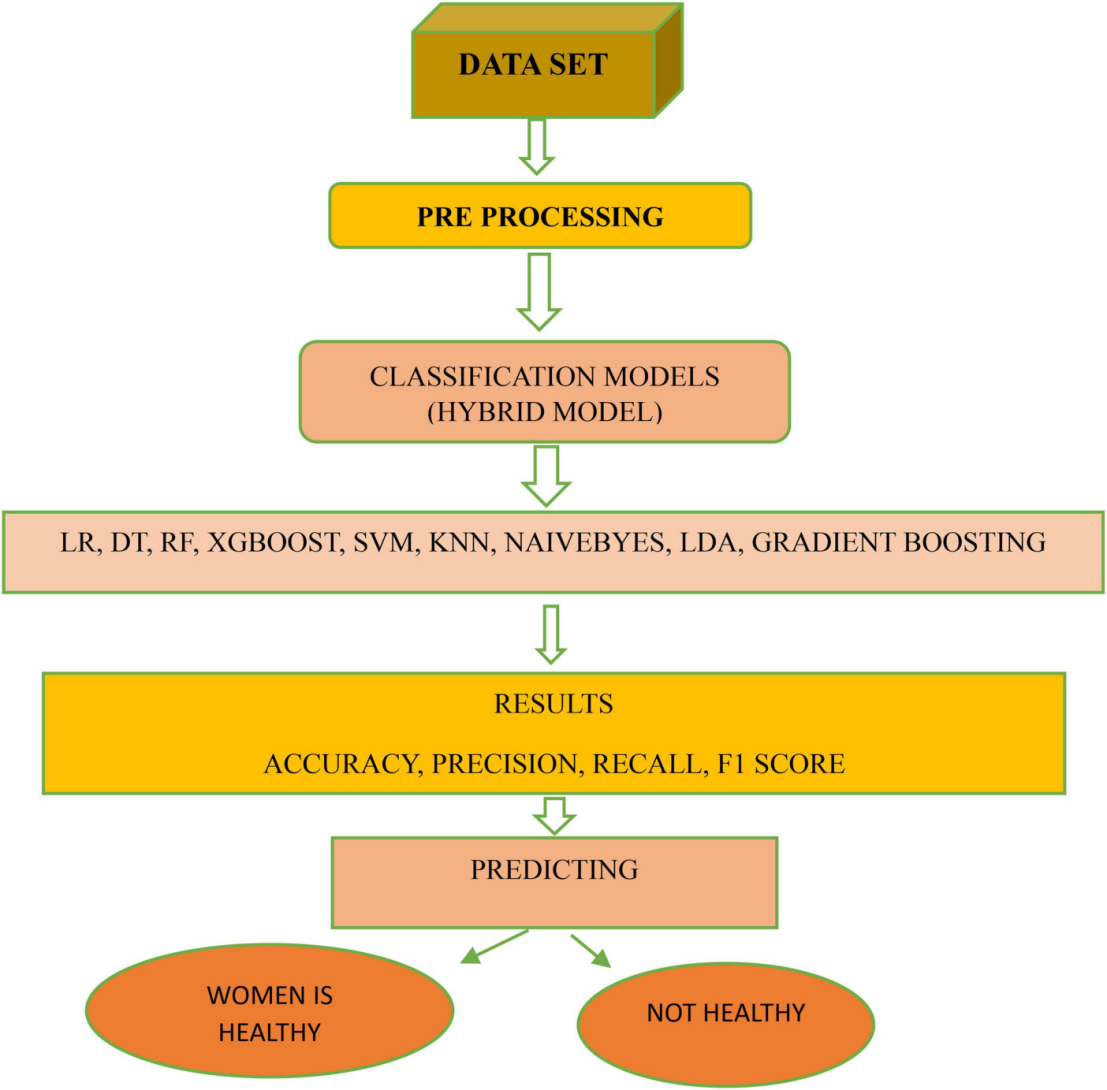
Proposed system.

To use ART such as IVF and IUI most successfully, the demographic profile of the woman must be considered. Even small lifestyle changes can have a majorly positive effect and lead to successful conception in infertile couples. To study the demographic profile of women, data were collected from married and unmarried women aged 25–60 . The data set comprises information about their daily lifestyle like sleep schedule, breakfast options, junk food, and exposure to hot environments. It also comprises data like technology use, alcohol consumption, smoking habits, and medicine use. Despite the proliferation of technology in the modern world, overuse of technology can lead to major health issues, one of which is infertility. Figure 1 demonstrates the proposed system, which involves steps taken to build the hybrid model.

### 2.2 Data preprocessing

1. To prepare the dataset for analysis and machine learning model training, the following data processing steps were performed: Removing Duplicate Values: duplicate or redundant values were removed through python and at certain points Power BI was also used to analyze the data.
2. Encoding Categorical Values: Different categorical data were converted to numeric data so that it could be easily used for analysis by different machine learning models.
3. Missing Values: Data were cleaned by removing null values so that the model could be trained effectively.
4. Normalizing Features: Data normalization was done to ensure that they were on a similar scale.
5. Splitting the Dataset: We divided the data, with 80% used for training and 20% for testing, to evaluate the performance of the hybrid machine learning model.

### 2.3 Correlation matrix

**Figure 2:**
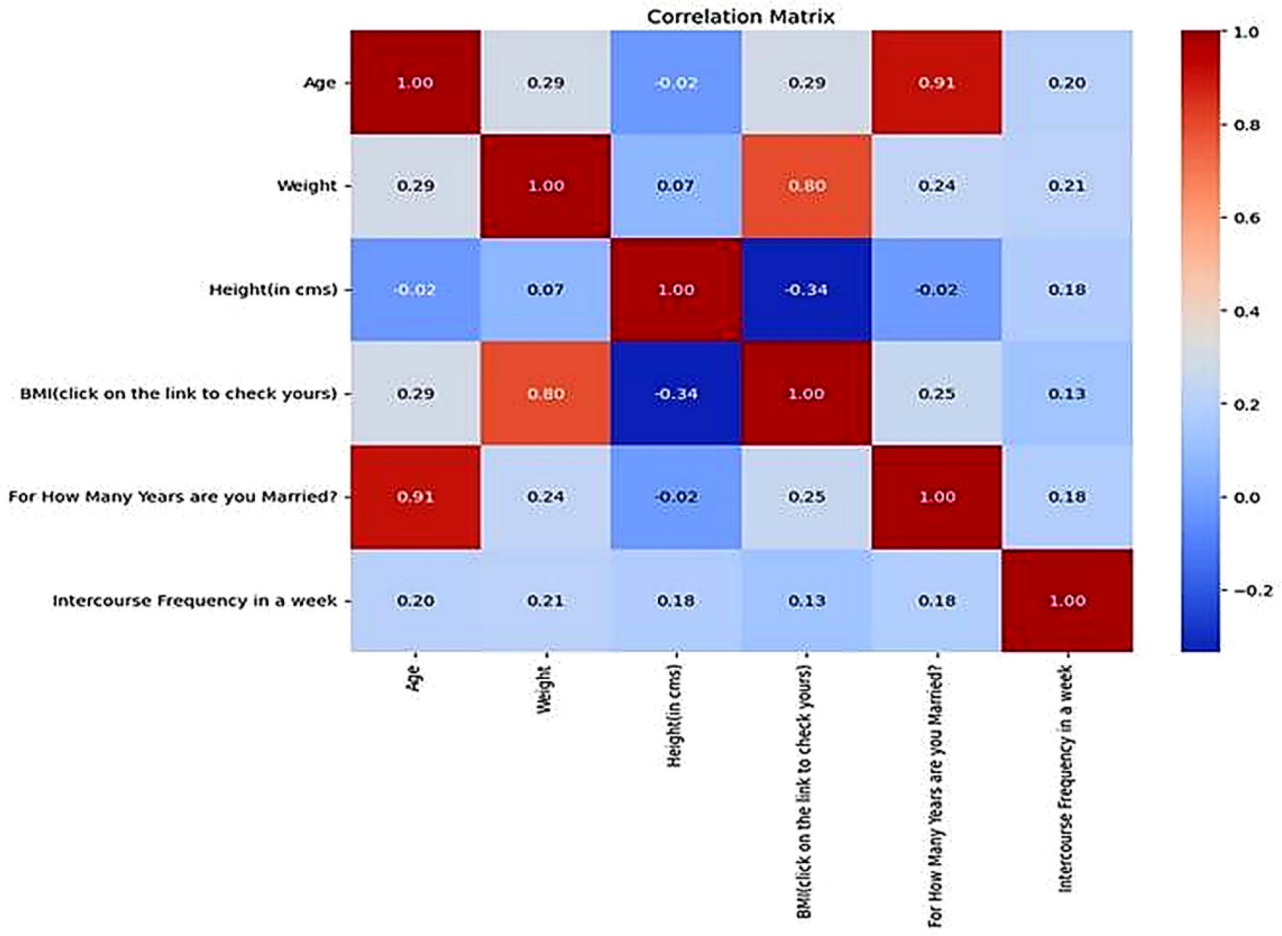
Correlation matrix.

A correlation Matrix was used to provide information related to the relationship between numerous categories. As Figure 2 shows, there is a strong correlation between age and years of marriage and weight and BMI. As age increases, egg quality reduces (7) Oocyte number and quality decrease with age. So, fertility decreases as age increases. Older women use more reproductive techniques compared to younger women (8). BMI is derived from weight and height. There is a moderate correlation between age and weight, age and BMI, and BMI and years married. Features like intercourse frequency per week is a unique feature that can give information about the reproductive health of a women (9). If women younger than 35 years of age are unable to conceive they are less likely to succeed using natural methods (10). Another study also supports the fact that egg quality correlates more strongly with maternal age than ovarian reserve count. Our research supports the fact that more frequent intercourse results in a higher probability of conception. There exists a relation between age, BMI, weight, height, and intercourse frequency for successful conception. Our research also states that to achieve successful pregnancy, a woman should understand her body, reduce her weight, maintain a healthy diet, and then must try in every cycle to achieve successful conception.

### 2.4 Model building

This research focuses on the demographic profiles of women. The dataset is partitioned into an 80% training set and a 20% testing set. The training dataset is used to train the model and the testing dataset is used to assess the model performance. Different Machine Learning Algorithms like Logistic Regression, Decision Tree, Random Forest, XGBoost, Support Vector Classification, KNN, Naive Bayes, and Linear Discriminant Analysis are used in a hybrid mode to predict health status. All the algorithms above are suitable for predicting health status, as the choice of algorithm plays an important role in health care sectors, particularly reproductive medicine. We used a bagging-based ensemble with majority voting in hybrid models built with different machine learning models. We used a heterogenous bagging-based ensemble, where multiple base learners were trained on bootstrap—resampled subsets of training data. Final results were predicted through majority voting, which improved robustness and reduced variance.

#### 2.4.1 Hybrid model 1 (logistic regression, decision tree, random forest, XGBoost, SVC, KNN, Naive Bayes, and linear discriminant analysis)

We trained a model with a total of 21 features from the dataset. We had built a hybrid modelling via ensemble learning by taking a combination of LR, DT, RF, XGBoost, SVC, XGBoost, Naïve Bayes, and Linear Discriminant Analysis. The ensemble accuracy achieved through the hybrid model was 70%. Model Dominance in ensemble as was using python: XGBoost: 10/10 (100.00%), SVM: 10/10 (100.00%), LDA: 10/10 (100.00%), Logistic Regression: 9/10 (90.00%), KNN: 9/10 (90.00%), Decision Tree: 8/10 (80.00%), Random Forest: 8/10 (80.00%), Gradient Boosting: 8/10 (80.00%), and Naive Bayes: 7/10 (70.00%).

#### 2.4.2 Hybrid model 2 (random forest, decision tree, logistic regression, KNN, SVC, and XGBoost)

This hybrid model was built by taking 21 features on the dataset and using supervised algorithms like Random Forest, Decision Tree, Logistic Regression, KNN, SVC, and XGBoost. The accuracy achieved was 80%. Model Dominance in ensemble was calculated using python: Logistic Regression: 10/10 (100.00%), Random Forest: 10/10 (100.00%), XGBoost: 10/10 (100.00%), SVM: 9/10 (90.00%), Decision Tree: 7/10 (70.00%), and KNN: 7/10 (70.00%).

#### 2.4.3 Hybrid Model 3 (Naïve Bayes, Logistic Regression, and Decision Tree)

This hybrid model was built using supervised models like Naive Bayes, Logistic Regression, and Decision Tree. The accuracy achieved was 60%. Model dominance in ensemble was calculated using python: Logistic Regression: 10/10 (100.00%), Decision Tree: 10/10 (100.00%), and Naive Bayes: 6/10 (60.00%).

#### 2.4.4 Hybrid model 4 (logistic regression, decision tree classifier, and linear discriminant analysis)

This hybrid model was built using supervised models like Logistic Regression, Decision Tree, and Linear Discriminant Analysis. The accuracy achieved was 90%. Model dominance in ensemble was calculated using python: Logistic Regression: 10/10 (100.00%), LDA: 9/10 (90.00%), and Decision Tree: 7/10 (70.00%).

Confusion Matrix refers to the table that shows how well the model performs by comparing actual and predicted values. There are four terms in the Confusion Matrix:

1. True Positive (TP): The model predicted the actual results (i.e., the model predicted a healthy woman was healthy). On the whole, TP means good detection.
2. True Negative (TN): The model predicts correctly, meaning TN is a safe approach.
3. False Negative (FN): The model says a woman is healthy but in reality she has some health issues.
4. False Positive (FP): The model predicts wrongly and is referred to as a missed case.

**Figure 3:**
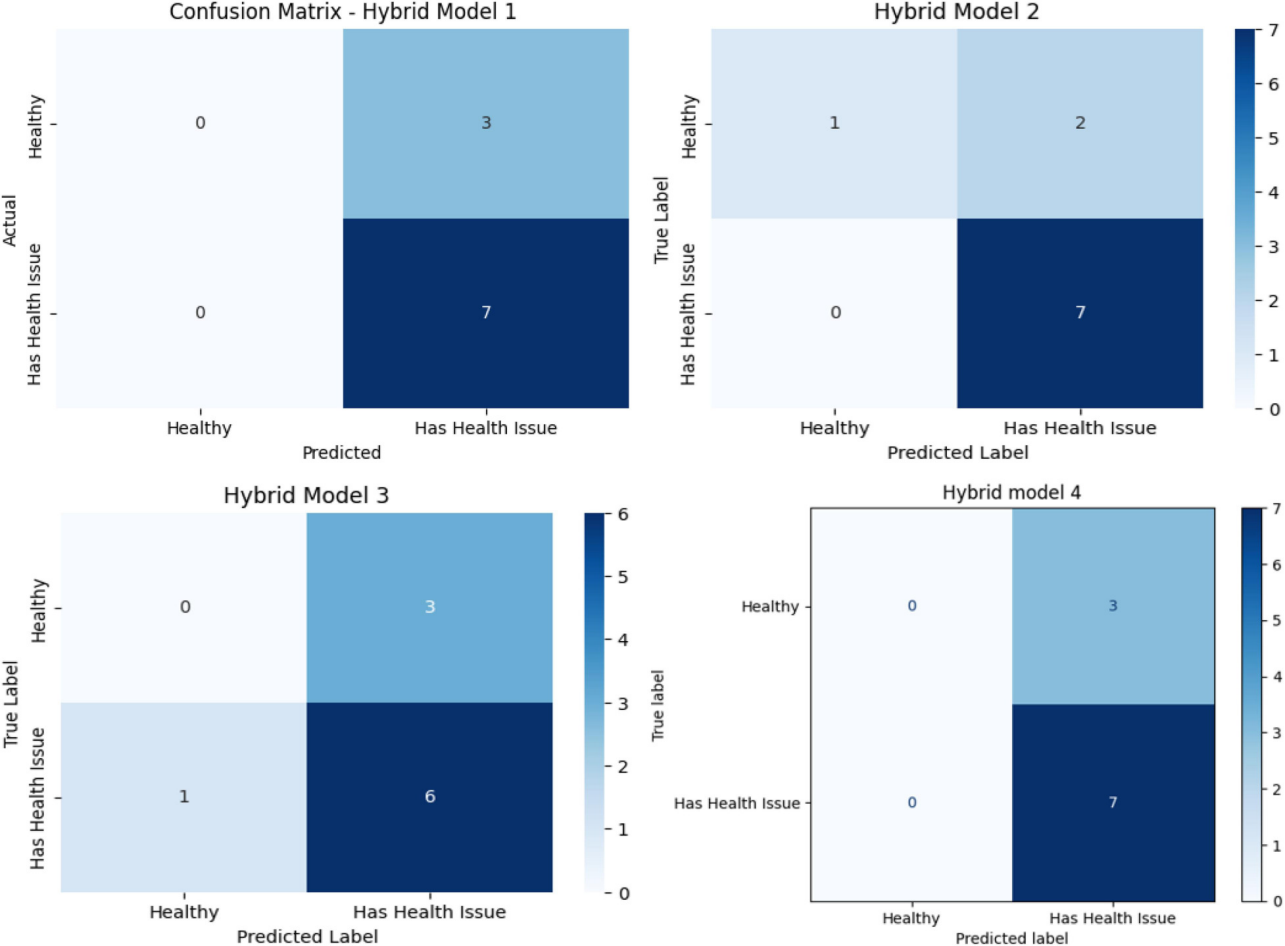
Confusion matrix.

Figure 3 provides the confusion matrix of the predicted four Hybrid Models using Supervised Learning Algorithms. Hybrid Model 1 predicted all the women who have health issues like PCOS and PCOD. Hybrid Model 2 predicted both the healthy and unhealthy women. We can say it is an improvement to model 1. Hybrid model 3 and 4 also performed well in comparison to other models. We can say from the results that models are more accurate with women with health issues because the primary data involved women with one or more health issues. From the data which ee have collected, around 96% of women had health issues like PCOD, PCOS, stress, and anxiety. According to the predictive model, this is attributed to poor dietary habits, lifestyle changes, neglected health, and the prolonged use of electronic devices such as laptops and mobile phones.

Hybrid models are also constructed using supervised learning and cross-validation techniques (including both k-fold and stratified cross-validation). The specific models developed are

1. Hybrid Model 1 (Naive Bayes, Random Forest, and SVM)

This hybrid model was built using supervised models like Naive Bayes, Random Forest, and SVM. The cross-validated Ensemble Accuracy was 75.56%.

2. Hybrid Model 2 (Logistic regression, KNN, and Linear Discriminant Analysis)

This hybrid model was built using supervised models like Logistic Regression, KNN, and Linear Discriminant Analysis. The cross-validated Ensemble Accuracy was 71.33%.

3. Hybrid Model 3 (Decision Tree, Random Forest, and XGBoost)

This hybrid model was built using supervised learning models like Decision Tree, Random Forest, and XGBoost. The cross-validated Ensemble accuracy was 81.56%.

The benefit of applying stratified fold is that we get a more reliable performance estimation as it reduces dependency upon a single train-test split and we obtain a better model. In our case, Hybrid Model 3 produced the best accuracy of 81.56%.

## 3 Result and discussion

The study was conducted on a laptop with Processor 11th Gen Intel(R) Core (TM) i3-1125G4 @ 2.00 GHz, 1997 Mhz, 4 Core(s), 8 Logical Processor(s), and 8 GB of RAM using Visual Studio code. The dataset containing 31 attributes was pre-processed to remove outliers and enhance model accuracy. The study used Supervised Machine Learning Models like KNN, Random Forest, Decision Tree, SVM, XGBoost, Naive Bayes, LDA, and Gradient Boosting algorithms. For every hybrid model, the performance matrix was also calculated. Of the date, 80% were used to train the machine and 20% were used to assess the model performance. The respective AUC score for Hybrid model 1 was 0.70, for Hybrid model 2 was 0.80, model 3 was 0.60, and for model 4 was 0.90; their corresponding accuracy was 70%,80%, 60%, and 90%, respectively. We also used the concept of cross fold that includes stratified and k fold validation. After applying cross fold, the following results were obtained. In hybrid Model 1, which uses naive bayes, Random Forest, and SVM, the accuracy achieved after k folds was 88.89%, average accuracy achieved after 5 folds was 77.78%, and accuracy achieved using stratified folds was 77.56%. In Hybrid Model 2, which uses models like Logistic Regression, KNN, and LDA, the accuracy achieved after k folds was 65.56% and through stratified folds was 65.33%. In Hybrid model 3, which uses Decision Tree, Random Forest, and XGBoost, the accuracy achieved after k folds was 75.33% and using stratified folds was 73.56%. Figure 3 specifies the confusion matrix of the predicted hybrid model 1, hybrid model 2, hybrid model 3, and hybrid model 4. Table 2 gives the evaluation matrix of predicted hybrid models. Table 3 provides a comparison of the predicted Hybrid models using k folds.

**Table 2:** Evaluation matrix results of different hybrid models.

Hybrid models	Accuracy	Precision	Recall	F1 score
Model 1(LR, DT, RF, XGboost, SVC, Naïve Bayes and Linear Discriminant Analysis)	70%	0.70	1.00	0.82
Model 2(Random Forest, Decision Tree, Logistic Regression, KNN, SVC and XGBoost.)	80%	0.78	1.00	0.88
Model3(Naive Bayes, Logistic Regression and Decision Tree)	60%	0.67	0.86	0.75
Model 4(Logistic Regression Decision Tree, Linear Discriminant Analysis.)	90%	0.70	1.00	0.82

**Table 3:** Cross validated ensemble accuracy.

Model	Combinations	Fold 1	Fold 2	Fold 3	Fold 4	Fold 5	Accuracy
Hybrid Model 1	Naive Bayes, Random Forest, and SVM	70.00%	70.00%	80.00%	80.00%	77.78%	75.56%
Hybrid Model 2	Logistic regression, KNN, and Linear Discriminant Analysis	50.00%	90%	90%	60.00%	66.67%	71.33%
Hybrid Model 3	Decision Tree, Random Forest, and XGBoost	80%	80%	80%	90%	77.78%	81.56%

The hybrid model trained with Logistic Regression, Linear Discriminant Analysis, and Decision Tree classifier performed the best, with an accuracy of 90%. The said model performs better because LDA can help improve model performance by minimizing the variance within each class. A model built with a combination of different supervised models along with LDA leads to better results and is widely used in medical diagnosis. The best performance of the hybrid model made using k folds with Decision Tree, Random Forest, and XGBoost was 81.56%. After analysis of the results, we can say that model built with DT, RF, and XGBoost reduces variance and can lead to better generalization on unseen data. We predicted that two Hybrid models—one with Ensemble learning using a combination of LDA, LR, and DT and one with k folds built with DT, RF, and XGBoost—would give 81.56% accuracy. Both Hybrid models aim to provide a demographic profile of women based on our collected data. We also applied cross validation to ensure more reliable prediction for model 3 trained using DT, Random Forest, and XGBoost, which achieved an accuracy of 81.56%.

## 4 Discussions

Based on the literature reviewed, no existing models have been constructed using primary data to predict women's health outcomes or focus significantly on their demographic profiles Our study focuses on women’s health, which is often neglected, with women being offered treatments whenever they are diagnosed with infertility.

Based on the ensemble learning results, Hybrid Model 4—comprising Logistic Regression, Decision Tree Classifier, and Linear Discriminant Analysis—achieved the highest performance with 90% accuracy. The research evaluated various machine learning algorithms/models to study the demographic profile of women using 21 features. Of all the hybrid models created with supervised machine learning algorithms, model 4 with LR, DT, and LDA performed best. We also applied Cross Validated Ensemble Accuracy where a model involving Decision Tree, Random Forest, and XGBoost performed best with an accuracy of 81.56%. We applied k folds as 5 folds to predict the results. After working with cross folds and ensemble learning, decision tree was a successful method in predicting the reproductive health of women. The benefit of applying Ensemble learning over single models is that it produces more reliable and stable predictions. It also solves the problem of overfitting and can easily handle bias variance trade-off as bagging reduces variance, boosting reduces bias, and stacking reduces both variance and bias. Our predicted model produced consistent outputs due to the use of ensemble learning. Our predicted model can be used to determine the reproductive health of women before they are prescribed ART methods like IVF/IUI/ICSI. To ensure more reliability, we have applied cross fold where our model trained using Random Forest, Decision Tree, and XGboost performed best with 81.56%. Our hybrid model trained using ensemble learning provides more accurate and robust results compared to the linear models. Women who are unable to conceive can work on their health; lifestyle change can improve the ability to conceive naturally. This means they can avoid the potential financial burden and mental difficulty of ART procedures.

### 4.1 Challenges

The data were collected using a Google form ,so there are chances of recall bias and reporting bias, but we have tried our best to ensure better results with our predicted model. The proposed hybrid model demonstrates predictive performance; it does not establish clinical efficiency. Our model was trained on real time data and so adds more value and may contribute to the field of reproductive medicines and ART.

## 5 Conclusion and future scope

In conclusion, this study examines the demographic and reproductive profiles of women to provide clinicians with a comprehensive understanding of a patient's health prior to prescribing ART procedures. Various Supervised machine Learning models like SVM, Naive Bayes, Decision Tree, random Forest, Logistic Regression, and LDA were used to construct the hybrid model and obtain accurate predictions. The research satisfies the research problem and gives valid predictions when testing was performed on hybrid models. It is essential that, before opting for ART procedures, women understand their bodily and reproductive health. This will certainly increase the chances of conception if they are prescribed ART procedures or mean they can conceive naturally through simple lifestyle changes.

Our model integrates with clinical practice, but validation with real-world results are crucial steps for the practical implementation of our model. Our model clearly shows that, before putting a woman on reproductive drugs, lifestyle changes should be performed to improve chances of natural conception.

Despite the promising results, the study has several limitations. First, the size of the dataset is relatively limited. We included only a particular age group of 25–60-year-olds. Second, although we tried our best to include the best models, the analysis was constrained to the available features and unmeasured clinical variables may affect predictability. Apart from all the limitations above, the hybrid model is promising and can be used to study to demographic profiles to further help clinicians to understand women’s health. Further, researchers can work on imaging data to understand the body in a more detailed manner and predict more robust results.

## Data Availability

The datasets presented in this article are not readily available because data were collected from women on the condition that they will only be used for this research. Requests to access the datasets should be directed to latika/latikaraheja01@gmail.com.
